# Aptasensors for *Staphylococcus aureus* Risk Assessment in Food

**DOI:** 10.3389/fmicb.2021.714265

**Published:** 2021-09-16

**Authors:** Ziqian Huang, Xin Yu, Qingli Yang, Ying Zhao, Wei Wu

**Affiliations:** ^1^College of Food Science and Engineering, Qingdao Agricultural University, Qingdao, China; ^2^Qingdao Municipal Hospital, Qingdao, China; ^3^Department of Anesthesiology, Qilu Hospital, Cheeloo College of Medicine, Shandong University, Qingdao, China

**Keywords:** *Staphylococcus aureus*, detection, aptamers, biosensors, aptasensors

## Abstract

*Staphylococcus aureus* (*S. aureus*) is the top ordinary pathogen causing epidemic and food poisoning. The authentication of *S. aureus* has great significance for pathologic diagnosis and food hygiene supervision. Various biosensor methods have been established for identification. This paper reviews the research progress of aptasensors for *S. aureus* detection, focusing on the classification of aptamer technologies, including optical aptasensors and electrochemical aptasensors. Furthermore, the feasibility and future challenges of *S. aureus* detection for aptamer assays are discussed. Combining aptasensors with nanomaterials appears to be the developing trend in aptasensors.

## Introduction

Foodborne pathogens caused by microorganisms are the main problem of food safety. *Staphylococcus aureus* (*S. aureus*), an anaerobic Gram-positive bacterium, is a common cause of foodborne intoxications (Hulme, 2017), with strong adaptability and the ability to tolerate a wide range of pH, temperature, and humidity. *S. aureus* strains produce one or more extracellular proteins, called staphylococcal enterotoxin (SE), which are composed of staphylococcal enterotoxin A (SEA), staphylococcal enterotoxin B (SEB), staphylococcal enterotoxin C (SEC), etc. (Principato and Qian, 2014). These extracellular proteins may inhibit the host’s immune response to *S. aureus*; hence, SE is thought to be the typical cause of food poisoning in humans after eating contaminated food. Infected people frequently develop gastrointestinal symptoms such as feeling sick, emesis, and diarrhea within hours. The disease is generally mild and usually resolves within 24–48 h of the onset of symptoms and rarely requires hospitalization. Foods susceptible to staphylococcal intoxication are usually meat, meat and egg products, milk (especially if animals are affected by mastitis), and baked goods (McMillan et al., 2016; Johler et al., 2018). In Spain, 21% of 940 food samples (milk, cheese, meat, baked goods, etc.) were reported to be positive for *Staphylococcus* spp. in 2016 (European Food Safety Authority and European Centre for Disease Prevention and Control, 2017). In 2017, a total of 4,600 animal specimens were collected in Italy; 28.8% of them were positive. Among these data, the incidence of sheep (37.4%) was significantly high (European Food Safety Authority and European Centre for Disease Prevention and Control, 2018). Salty foods such as ham are also implicated by the ability of *S. aureus* that grew with low moisture activity (Qi and Miller, 2000). It is critical to detect efficiently and to prevent the occurrence of the disease since *S. aureus* has become a kind of pathogenic bacteria that caused serious harm to food safety. The risk assessment of foodborne pathogens can quickly and effectively assess the pathogenic factors of different types of foodborne diseases, such as bacterial food poisoning, by constructing early outbreak prediction model. Food safety risk assessment, especially microbial risk assessment (MRA), plays an important role in ensuring food safety and controlling foodborne diseases. An accurate and reliable risk assessment process is essential for people’s health and safety.

*Staphylococcus aureus* biofilms can form physical barriers that affect the spread and distribution of antibiotics; bacteria are encapsulated in the extracellular biofilm matrix and arranged in multiple layers, which can develop resistance to antimicrobial agents and host immune systems by damaging the action of phagocytes (Prenafeta et al., 2014). The occurrence of the methicillin-resistant *Staphylococcus aureus* (MRSA) is on account of the excellent capacity of *S. aureus* to suit antibiotics. Enterotoxin-producing MRSA can also act as a foodborne pathogen under growth conditions favorable for enterotoxin production. MRSA has long been recognized as a major pathogenic factor in human healthcare-related infections (HA-MRSA) (Sergelidis and Angelidis, 2017). MRSA strains have been implicated in community-associated infections (CA-MRSA) in many countries (Deurenberg et al., 2007). It has been reported that MRSA transported on poultries and domestic animals are called LA-MRSA (Macori et al., 2017). Up to now, the presence of LA-MRSA in live domestic animals, wild animals, fresh foods, and ready-to-eat foods has been demonstrated in a number of studies (Sieber et al., 2018; da Silva et al., 2020). A study in Greece examined 367 samples (36 bulk tank milk, 19 milk dairy products, 72 humans, 185 animals, and 55 pieces of equipment), of which 57.8% of the samples tested positive for *S. aureus* (Papadopoulos et al., 2018). Identical conclusions were obtained from studies that people, animals, and the surrounding environment may be related to MRSA contamination in the dairy production chain (Tegegne et al., 2019).

Tuerk and Gold (1990) established SELEX technology, which successfully screened synthetic oligonucleotides with high affinity and specificity from RNA library. The SELEX process is implemented in DNA library, and single-stranded DNA (ssDNA) was prepared by thermal deformation of DNA library. Generally, the target is fixed on the magnetic beads as the selection object, and then the ssDNA aptamer of the target is selected *in vitro*. The magnetic beads modified with the target are co-incubated with the DNA library, and the unbound or weakly bound ssDNA is discarded after magnetic separation. The ssDNA combined with the magnetic beads is eluted and collected as the template for polymerase chain reaction (PCR) amplification. After PCR amplification, a new ssDNA library is formed, which will be SSco-incubated with magnetic beads in the next round of screening. In the screening process, negative selection and inverse selection are combined to reduce the enrichment of non-specific ssDNA during selection. The incubation, elution, and amplification steps are repeated continuously, with the increase of screening times, the incubation time will be shorter; ultimately, the ssDNA aptamer of the target will be finally obtained. Multiple SELEX screenings give the aptamer a higher specificity with more stable affinity than antibodies. The concept of aptamer was first proposed by Ellington and Szostak (1990). So far, many aptamers have been developed for *S. aureus* and its toxins (DeGrasse, 2012; Baumstummler et al., 2014; Huang et al., 2014, 2015).

By virtue of its three-dimensional structure, aptamer is highly selective in binding to targets and has high affinity and strength specificity similar to antigen–antibody reaction, which can detect targeted pathogens in complex food samples. Biosensors consist of recognition elements and sensors. The signal of the sample is amplified by the biometric element, and the sensor transforms the biometric signals into measurable signals. Aptamer-based multi-class materials can be used as signal amplifiers to establish aptasensors, which can obtain higher sensitivity and is more suitable for rapid detection of pathogens in the field, and have become a new method for risk assessment of pathogens.

## Available Methods for Detecting *S. aureus*

**FIGURE 1:**
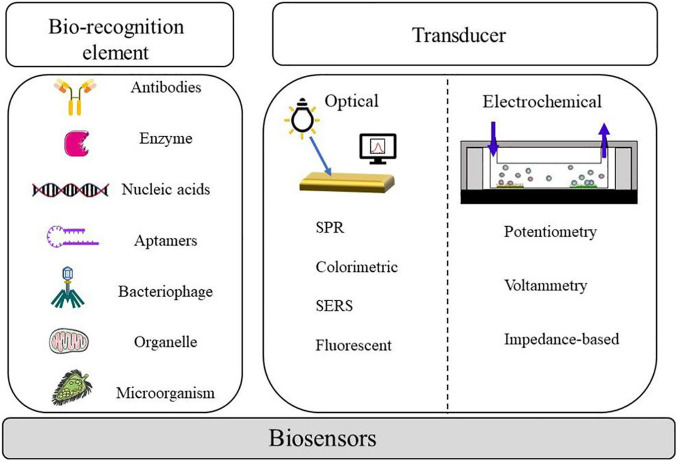
Components and measurement methods associated with biosensors for pathogen detection.

In the past two decades, foodborne pathogen detection by conventional ways mostly relies on the culture and identification of microorganisms, which are accurate and reliable; however, it remains challenging due to laborious duty and a long period of experimental operation. With the development of molecular detection technology, PCR has been gradually applied to foodborne pathogenic bacteria detection. However, owing to its inability to distinguish between dead and alive bacteria as well as the fact that the experiment is prone to interference, it is difficult to satisfy the requirements of actual detection (Tao et al., 2020). Compared with traditional antibodies, aptamers, which act as a new molecular recognition element, have the advantages of a wide range of target molecules, fabulous stability, long storage life, and high specificity (Gopinath et al., 2016). Biosensors based on nucleic acid aptamer have proved promising for detecting foodborne pathogens. The aptamer biosensor made by combining the biosensor technology not only has the characteristics of high specificity, strong affinity, easy modification, and good stability but also maintains the advantages of rapid response, simple operation, and low cost of the biosensor. Figure 1 shows a diagram of representative components and techniques that can be integrated into a biosensor in order to detect the pathogens.

Conventionally, the detection methods of foodborne pathogens will be further discussed below, which can be systematized by culture-based, nucleic acid-based and immune-based methods. The culture method is currently the most widespread and sophisticated test method and is generally recognized as the gold standard for microbiological analysis of foodborne pathogens. The culture-based method relies on the cultivation of microorganisms on Agar plates to form visible colonies; next, the colonies are subjected to a standard biochemical identification that provides qualitative or quantitative analysis of pathogen bacteria in food samples. This method remains the preferred method for numerous food testing laboratories since it is 10-cent and easy to use. However, preliminary results can take 2–3 days, while identification of specific pathogenic microorganisms can take more than a week (Zhao et al., 2014). Because of this, it is incapable to handle food safety emergencies.

Nucleic acid-based methods manipulate the DNA or RNA sequence of a target pathogen by detecting specificity. In recent years, it is generally accepted that nucleic acid-based methods are divided into several categories, including simple PCR, multiplex polymerase chain reaction (MPCR), quantitative polymerase chain reaction (qPCR), and loop-mediated isothermal amplification (LAMP). The most widely used nucleic acid amplification method to detect pathogens is PCR. This technique was first reported by Saiki et al. (1985). The principle is that two nucleic acid chains from different sources have complementary base sequences, which can specifically bind to form molecular hybridization chains (Ramesh et al., 1992).

Polymerase chain reaction can detect many toxin genes of *S. aureus* in a short time. The very first time PCR was utilized to detect *S. aureus* was reported in 1991 (Wilson et al., 1991). After adding the total DNA of the object to be tested, the sequence of the target gene of SEB is amplified by PCR or real-time fluorescence PCR, and quantitative monitoring can be conducted by electrophoresis bands or fluorescence intensity. Martinon and Wilkinson (2011) established a low-cost, SYBR Green based double-chain real-time PCR for simultaneous detection of *Listeria monocytogenes* and *S. aureus* in food. Detection limit was 2 CFU/g in raw meat containing *S. aureus* (Martinon and Wilkinson, 2011). However, PCR-based tests used alone are unable to provide any indication of the cell viability being examined because they do not distinguish DNA from living and dead cells (Foddai and Grant, 2020). The specificity of the PCR technology depends on the specificity of the primer and template DNA binding; without increasing the fungus, it can detect *S. aureus* directly from the gene level, and more accurate, rapid, and more sensitive (Smith and Osborn, 2009) detection has been successfully carried out in a variety of pathogens, but the price is higher.

Multiplex PCR provides faster detection by simultaneously amplifying multiple gene targets compared to simple PCR. Tao et al. (2020) established a common primer-mediated MPCR technique for non-qualitative screening of 11 common foodborne pathogens. Park et al. (2006) used MPCR to detect *S. aureus* in kimchi, and the detection limit was 260 CFU/ml. qPCR, also known as real-time PCR, is a way that continuously monitors the production of PCR products throughout the reaction process, providing rapid, simultaneous amplification and gene detection (Valderrama et al., 2016). LAMP is a DNA isothermal amplification technique proposed by Notomi et al. (2000). This research employed a DNA polymerase and a set of primers specially designed to identify six different sequences in the target DNA and does not require a cyclic process such as transgenesis during the whole process. It is a new DNA amplification method, which is simple, quick, and highly specific (Srividya et al., 2019), and has the possibility of replacing the PCR method. At the same time, in the actual detection work, it was found that PCR technology has high requirements for primers, system establishment, and annealing temperature screening; high requirements for target fragment screening; and high sampling standards. Immunoassay method for detecting foodborne pathogens is based on the antigen–antibody union. It is used to quickly detect pathogens, which have not yet been realized by other conventional methods. The most frequently used immunoassay method is enzyme-linked immunosorbent assay (ELISA), which is widely applied to the detection of foodborne pathogens, fungal toxins, and bacterial toxins. While short detection time and high sensitivity are required compared to traditional culture-based methods, ELISA falls short of being able to detect pathogens in real time (Umesha and Manukumar, 2018). Sandwich ELISA is a modified ELISA that uses two antibodies against a single antigen (Priyanka et al., 2016). The usage of sandwich ELISA for rapid foodborne pathogen detection has been studied, and the sensitivity and specificity of sandwich structure are much higher than before. The presence of the bacteria can also be proved by testing for enterotoxins. Immunoassay has become the main tool for rapid detection of harmful toxigenic bacteria due to its high specificity for toxicity. Nouri et al. (2018) designed a new kit for the detection of SEA in milk, with a detection time of about 15 min and a sensitivity of 15.6 ng of toxin. For instance, Sun et al. (2020) developed a sandwich chemiluminescent immunoassay (CLIA) to detect SEB, an anti-SEB monoclonal antibody was used as the capture antibody, and Nb37-ALP was used as the detector antibody; the detection limit was 1.44 ng/ml. In another sandwich ELISA assay, nano-antibodies acted as capturing antibodies, and the detection antibodies were acted by phage nanoantibodies with amplified signal properties. Under the optimal cases, the quantitative range of this method was 1–512 ng/ml, and the detection limit was 0.3 ng/ml (Ji et al., 2020). In actual detection, this method is highly dependent on equipment; secondly, antibody preparation is difficult and it is prone to false positives; finally, the experiment has certain requirements such as the professional level of the experimenter. Obviously, the methods mentioned above are not sufficient, and most of them are longstanding and laborious jobs. To a large extent, aptasensor detection can alleviate the problems of the above methods. Table 1 summarizes the advantages of conventional methods and aptasensors for *S. aureus* detection.

**TABLE 1:** Comparison of conventional methods and aptasensors for detection of *S. aureus*.

**Detection methods**	**Basic principle**	**Advantages**	**Limitations**	**Detection limit (CFU/ml)**	**Assay time**
Culture-based methods	Traditional culture is the growth of pathogens in the culture medium and the formation of visible colonies	Gold standard Cost-effective Simple	Time-consuming Complex operation	>10^4^	7 days
Nucleic acid-based methods	DNA or RNA sequences of target pathogens were manipulated by specific detection	Rapid Specific Sensitive	CostStandardized material Specialized experiment	10^3^–10^4^	10–24 h
ELISA	The basic principle is the combination of the antibody with the antigen followed by the detection of the antigen–antibody complex	Highly specific Rapid Cost-effective Automatic machine application	Poor stability Existing cross-contamination Equipment required	10^3^–10^5^	3–10 h
Aptasensors	The detection is based on the high-affinity and high-specificity binding of the secondary or tertiary structures formed by single nucleotides	Wide target range High affinity and specificity Good thermal stability Long storage life Stable properties Flexible Low molecular weight	Limited conformational diversity Time-consuming and low success rate of SELEX process	10^1^–10^5^	0.5–3 h

## Principles of Aptasensors for *S. aureus* Detection

Nucleic acid-based aptamers are a ssDNA fragment or short RNA sequences that are obtained by separation of nucleotides synthesized *in vitro* in libraries using SELEX. They have characteristics that can be easily synthesized *in vitro*, simple to modify, and can be designed flexibly in a sequence. Compared with traditional antibodies, aptamers have more advantages. As a new molecular recognition element, aptamers can recognize not only single molecules like protein and nucleic acid but also large molecular complexes such as cells, bacteria, microorganisms, and viruses. It has a wider range of target molecules (proteins, nucleic acids, parasites, bacteria, cells, viruses, etc.) and a higher affinity than antigen–antibody reaction; the molecular weight is about 20–100 bp, which makes it easier to enter the cell; the preparation process does not require antibody immunity; and animal experiments can be synthesized *in vitro* for subsequent experiments. Stable properties and longer storage life make aptamers the ideal experimental material. Massive aptasensors have been logically designed and miscellaneous techniques, including optical and electrochemical aptasensors, have been used and combined to acquire gratifyingly detectable signals. After screening the nucleic acid of foodborne pathogens, the detected signals should be further converted into recognizable output signals. The nucleic acid aptamer can perform signal output through the biosensor, fix the nucleic acid aptamer on the substrate of the biosensor, and transform the chemical, physical, electrical, or optical changes in the adsorption process into detectable signals through sensing technology. This paper introduces the application of these sensors in the detection of nucleic acid aptamer in foodborne pathogenic bacteria.

### Optical Aptasensors

With the advantages of high sensitivity, speediness, and specificity, the optical sensor has been widely used in *S. aureus* detection. It is commonly known that optical aptasensors are classified into surface plasmon resonance (SPR), colorimetric aptasensors, surface-enhanced Raman spectroscopy (SERS), and fluorescence (Rubab et al., 2018). Optical biosensors consist of a biological recognition layer, a transducer, and amplification. Various optical-based aptamer sensors that have emerged in recent years are summarized in Table 2.

**TABLE 2:** Optical-based aptasensors for detect *S. aureus*.

**Advantages**	**Limitations**	**Detection methods**	**Detection limit**	**Linear range**	**References**
Worthy sensitivity Label-free detection Quantification	Limited detection of the whole cell Requires a relatively large equipment Complex analysis system	SPR	10^6^ CFU/ml	10^5^–10^8^ CFU/ml	Wang et al., 2019
Simple and rapid Portability Cost-effective	Low sensitivity Limited quantification	Colorimetry	9 CFU/ml	10–10^6^ CFU/ml	Yuan et al., 2014
		Colorimetry	10 CFU/ml	10–10^6^ CFU/ml	Yao et al., 2020
		Colorimetry	81 CFU/ml in PBS	10^2^–10^7^ CFU/ml	Yu et al., 2020
		Colorimetry	1.5 × 10^7^ cells/ml	1.5 × 10^7^–5.3 × 10^7^ cells/ml	Lim et al., 2021
Superior sensitivity Able to characterize more details Label-free detection	Finite quantification capability Complicated analysis spectrum	SERS	13 CFU/ml	4.3 × 10–4.3 × 10^7^ CFU/ml	Zhu et al., 2021
		SERS	35 CFU/ml	10^2^–10^7^ CFU/ml	Zhang et al., 2015
		SERS	3 cells/ml	10^2^–10^7^ cells/ml	Pang et al., 2019
		SERS	1.5 CFU/ml	10–10^7^ CFU/ml	Gao et al., 2017
High sensitivity, stable Simultaneous detection Nanomaterials applications	Requires pretreatment Complex operation steps	Fluorescence	1.7 CFU/ml	7–7 × 10^7^ CFU/ml	Yu et al., 2017
		Fluorescence	64 CFU/ml	10^2^–10^7^ CFU/ml	Lu et al., 2020
		Fluorescence	7.6 × 10^2^ cells/ml	6.0 × 10^2^–6.0 × 10^5^ cells/ml	He et al., 2014

#### SPR Aptasensors

Surface plasmon resonance has the benefits of no mark detection, real-time supervision of the dynamic process of biological reaction, and non-destructive detection. SPR is the surface plasma produced by light at the interface of two kinds of dielectric constant materials to reduce the intensity of reflected light. Kinetic and equilibrium analysis of the presence of SPR provides access to characterize molecular interactions, for instance, the aptasensor binds to the analyte, the mutual effect between the antibody and the antigen, and the characterization of the receptor (Damborsky et al., 2016). The limit of detection (LOD) of SPR is based on several factors, such as the molecular weight of the target probe, optical properties, and the affinity of the probe (Nguyen et al., 2015). However, the size of pathogenic bacteria can interfere with some measurements, and the detection limit is often too high. Wang’s group assembled aptamers on a gold substrate mediated by polyadenine. The designed aptasensors can only show an SPR signal at concentrations of *S. aureus* greater than 1 × 10^6^ CFU/ml (Wang et al., 2019). The aptamer was applied to detect *S. aureus* in milk by resonance combination with localized surface plasmon resonance (LSPR). It should be pointed out that the LOD of aptasensors was 10^3^ CFU/ml, and the analysis time was only 120 s (Khateb et al., 2020).

Colorimetry has been widely used due to the following reasons: low cost, simple, practical, fast, and portable diagnosis. There is no need for an analytical device to easily and immediately verify the presence of pathogens in samples based on color variations (Song et al., 2011). Metal nanoparticles, such as gold and silver, are the subject of attention in aptasensors because of their optical properties related to size and distance. Chang et al. (2016) reported a duplex detection method based on aptamer and gold nanoparticles (AuNPs), which can accurately identify *S. aureus* from common pathogens. Using AuNPs as an indicator, the bacteria were first incubated with antagonistic *S. aureus* aptamers, and then aptamers were inserted into AuNPs to avoid the interaction between bacteria and AuNPs. When salt was added, AuNPs that were bound to the bacteria remained red, while those that were not turned blue (Chang et al., 2016). Yuan and coworkers developed colorimetric aptasensors for *S. aureus* based on AuNPs using tyramine signal amplification (TSA) technology. The method has a detection sensitivity of up to 9 CFU/ml, with a linear range of 10–10^6^ CFU/ml (Yuan et al., 2014). A colorimetric immunoassay is adopted based on immuno-magnetic and signal amplification of AuNPs etching to enhance the activity of peroxidase for *S. aureus* detection. IgY-Fe_3_O_4_/Au nanocomposite was regarded as the capture probe; at the same time, aptamer-AuNPs were used as the signal amplifier, and the *S. aureus* can be lightly caught by the naked eye at 10 CFU/ml and a linear range of 10–10^6^ CFU/ml (Yao et al., 2020). Yu et al. (2020) developed a colorimetric aptasensor for high-throughput detection of *S. aureus* catalyzed by aptamers and the dsDNA–SYBR Green I (Sg I) complex. In addition, this method can directly detect *S. aureus*, and the LOD in PBS buffer was 81 CFU/ml and the detection time was 5.5 h (Yu et al., 2020).

#### Surface-Enhanced Raman Spectroscopy Aptasensors

Surface-enhanced Raman spectroscopy is a kind of common sensing technology, which involves resonance Raman effect excited by plasmon. When molecules are attached to metallic surfaces, like silver nanoparticles and AuNPs, the light scatter of molecules increases. The mechanism of SERS can be divided into electromagnetic field enhancement and chemical mechanism (CM), which is due to the particular interaction of adsorbed substances between metal surfaces and molecules (Langer et al., 2019). It is generally believed that the electromagnetic field on the metal surface can be expressively raised due to plasma excitation because the electromagnetic mechanism provides most of the enhancement (Payton et al., 2014). Zhu et al. (2021) assembled a *S. aureus* aptasensor using SERS technology based on an aptamer functionalized polydimethylsiloxane (PDMS) membrane. The aptamer was fixed on the AuNPS-PDMS membrane by Au-S. The gold-core silver-shell nanoflower (Au@AgNFS) revised by mercaptobenzoic acid (4-MBA) and aptamer was used as the signal probe. A sandwich structure used is for taking substrate target-signal molecular probes. Under optimized experimental conditions, LOD is 13 CFU/ml. The linear range of this experiment was 4.3 × 10 to 4.3 × 10^7^ CFU/ml (Zhu et al., 2021). Zhang et al. (2015) used Raman molecule-modified AuNPs and aptamers as signal probes. Fe_3_O_4_ magnetic AuNPS (Au-MNP) immobilized with aptamers were designed to capture *S. aureus*. Under optimal conditions, the LOD was 35 CFU/ml (Zhang et al., 2015). A magnet SERS biosensor was proposed based on the double recognition of pathogens by aptamers and antibiotics. Fe_3_O_4_@Au magnetic nanoparticles (Au-MNPs) modified with aptamers were compounded by bacteria-specific magnetism and SERS active substrates, and vancomycin-labeled SERS (Au@MBA) was intended for sensitive quantification of pathogens. *S. aureus* in real samples, such as milk and orange juice, were detected; the LOD was 3 cells/ml, and the detection of the aptasensor reported ranged from 10 to 10^7^ cells/ml (Pang et al., 2019). Zhang et al. (2018) first reported a dual vancomycin and aptamer identification of a sensitive SERS platform, with *Escherichia coli* (*E. coli*) and *S. aureus* as target bacteria, and, at the same time, detected 20 kinds of pathogenic bacteria. To sum up, the LOD was shown to be 20 cells/ml, and *S. aureus* was in the range of 20–10^5^ cells/ml (Zhang et al., 2018). It is generally accepted that SERS has overcome the shortcomings of Raman spectroscopy, such as flat Raman signal, poor LOD, and photobleaching (Smolsky et al., 2017).

#### Fluorescent-Based Aptasensors

Fluorescence has become one of the most commonly used sensing ways for the analysis and detection of low-concentration analytes because of its high sensitivity, high efficiency, and simple and rapid analysis. Fluorescence can be classified as labeled and unlabeled. Labeled fluorescence requires at least one chromophore or fluorescent cluster, and the typically labeled fluorescence assay is Förster (fluorescence) resonance energy transfer (FRET) detection. The realization of fluorescence signal mainly depends on the interaction between a non-radiative energy long-range dipole and a dipole used to transfer from the donor to the recipient, called FRET; there are generally two sensing strategies, namely, a signal on and signal off. Yu et al. (2017) proposed a dual recognition of *S. aureus* using vancomycin and aptamer nucleic acid based on a bimolecular affinity FRET platform. The donors and the receptors are, respectively, gold nanoclusters that function with vancomycin and aptamer-modified AuNPs, under optimal detection conditions; using this approach, the linear range for monitoring *S. aureus* was 20–10^8^ CFU/ml, with the LOD as low as 10 CFU/ml (Yu et al., 2017). Tao et al. (2021) designed a one-step FRET assay for *S. aureus* detection. With aptamer-modified quantum dots (QDs) as donors and antibiotics-modified AuNPs playing the role of acceptors, the detection time was 1 h, and the detection linear range was 10–5 × 10^8^ CFU/ml; the LOD in food samples (milk and orange juice) was 100 CFU/ml (Tao et al., 2021). Lu et al. (2020) introduced the protective binding influence between aptamers and targets, to construct the aptasensors, thus avoiding the optimization of the aptamer probe sequence. Sensitive detection of *S. aureus* was achieved (LOD was 64 CFU/ml, and the dynamic range was 10^2^–10^7^ CFU/ml). The method mentioned above can be used for high-precision quantification of *S. aureus* in tap water, milk, and pork (Lu et al., 2020). He et al. (2014) designed to immobilize the aptamer of *S. aureus* on fluorescent silica nanoparticles to generate Aptamer/FSiNPs. The experiment is described as follows, in a nutshell. Firstly, the sample is grown with the self-assembly, and then the sample was stained with DNA dye and finally detected by two-color flow cytometry. Using this approach, the aptasensor had a LOD of 1.5 × 10^2^ cells/ml in buffer and 7.6 × 10^2^ cells/ml in spiked milk (He et al., 2014). Based on carbon dots (CDs) and gold nanoparticles, Yao et al. (2021) established a one-step fluorescence method for *S. aureus* detection. When *S. aureus* occurs, the fluorescence signal is turned off according to the aptamer that preferentially binds to the pathogen phenomenon. The LOD of this unlabeled method was 10 CFU/ml, and the linear range was 10–10^6^ CFU/ml (Yao et al., 2021).

Molecules capable of fluorescing sensing are usually carried by structures called fluorophores and receptors, and in some systems, these two structures can be combined. A method for the detection of *S. aureus* fluorescence based on a molecular beacon (MB) and chain displacement target cycle has been developed by Cai et al. (2019). The results revealed that the detection range of *S. aureus* was 80–8 × 10^6^ CFU/ml, and the LOD was 39 CFU/ml (Cai et al., 2019). Hundreds of studies heretofore have focused on nanomaterials; a large number of nanomaterials such as QDs, up-conversion nanoparticles (UCNPs), CDs, AuNPs, graphene oxide (GO), and carbon nanotubes (CNTs) have been applied in fluorescence biosensors. High-sensitivity and multiplex methods have been established to detect simultaneously and specifically three pathogens, using polychromatic UCNPs as markers and aptamers as molecular recognition elements. Under optimum conditions, the bacterial concentration was linearly correlated with the luminescent signal in the range of 50–10^6^ CFU/ml. The LOD of this work was found to be 25 CFU/ml for *S. aureus* (Wu et al., 2014). Shrivastava et al. (2018) demonstrated a culture-free, rapid, quantitative method to detect *S. aureus* based on a smartphone. Marked *S. aureus* are captured by a magnet in a box where light-emitting diodes act as a source of excitement and then smartphone cameras are used to create a fluorescent image. The method allows the detection of *S. aureus* directly from peanut milk samples for 10 min, with a minimum detection concentration of 10 CFU/ml (Shrivastava et al., 2018).

### Electrochemical Aptasensors

**FIGURE 2:**
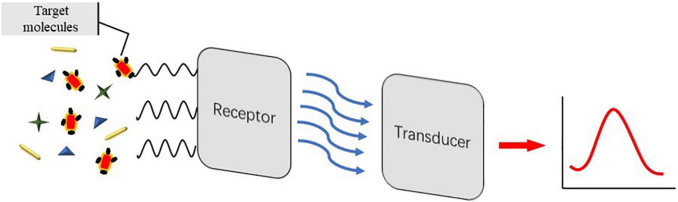
Schematic illustration of detection principle of electrochemical aptasensors.

Electrochemical biosensors can be widely used in pathogen detection for the safety of food and drinking water, medical diagnosis, environmental monitoring, and biological threats due to their wide variety (Yang et al., 2018). Electrochemical biosensors that can be used for real-time detection, with high specificity and no contamination, have become a bioanalytical method for clinical diagnosis of proteins in point-of-care systems (Chikkaveeraiah et al., 2012). As can be seen from Figure 2, the principle of electrochemical detection is expressed. Electrochemical aptasensors combined with a variety of nanomaterials (CNTs, graphene, GO, etc.) have been widely popularized in food and clinic (Ravalli et al., 2016; Wang et al., 2016; Pourakbari et al., 2019). Electrochemical aptasensors utilize electrodes as the transduction element and aptamers as the biometric identification element to convert signals into electrochemical signals. Ceramic electrodes, metal electrodes [Au and platinum (Pt)], polymer materials electrodes, and carbon electrodes have been widely used as electrodes (Amiri et al., 2020). The following three methods representing electrochemical transducer detection will be introduced: potentiometry, voltammetry, and impedimetry. Table 3 compares the electrochemical detection of *S. aureus* based on aptasensors.

**TABLE 3:** Comparison of analytical features of electrochemically related aptasensors for *S. aureus* detection.

**Detection methods**	**Working electrode**	**Detection limit**	**Linear range**	**References**
Potentiometry	GO/rGO aptasensor electrode	1 CFU/ml		Hernández et al., 2014
Potentiometry	Aptamer/graphene interdigitated gold electrode	41 CFU/ml	4.1 × 10–4.1 × 10^5^ CFU/ml	Lian et al., 2015
Impedimetry	Glassy carbon electrode	1 CFU/ml	1.2 × 10^1^–1.2 × 10^8^ CFU/ml	Ranjbar and Shahrokhian, 2018
Voltammetry	Carbon electrode	0.21 fM	5.0–500.0 fM	Mousavi Nodoushan et al., 2019
Chronocoulometry	Modified gold electrode	3 pg/ml	0.05–100 ng/ml	Chen et al., 2019

#### Aptasensor Detection Based on Potentiometry

Potentiometry, also known as amperometry, measures the potential by applying a current. One advantage of this method is the ability to use a low-cost measuring instrument. Hernández et al. (2014) synthesized a potentiometric aptasensor using graphene electrodes modified on carbon rods and an aptamer attached to graphene, which can catch a single CFU/ml of *S. aureus*. Lian et al. (2015) have developed a new piezoelectric sensor that connects *S. aureus* aptamers with gold electrodes, using aptamers as a recognition element. Using 4-mercaptobenzene-diazonium tetrafluoroborate (MBDT) as a molecular crosslinking agent, graphene was chemically bonded to the interdigital gold electrode (IDE) of a series of electrode piezoelectric quartz crystals (SPQC). At the time when *S. aureus* appears, the aptamer falls off the surface of graphene (Lian et al., 2015). Cai et al. (2021) designed an electrochemical method for *S. aureus* detection of three-helix molecular switches. An aptamer modified on the magnetic bead was used to capture the pathogens and release the complementary strand cDNA. In the next step, the gold electrode that modified the triple helix structure controls the release and shutdown of the signal. The system was able to detect water and honey samples, the LOD was 8 CFU/ml, and the linear range was from 30 to 3 × 10^8^ CFU/ml (Cai et al., 2021).

#### Aptasensor Detection Based on Voltammetry

**FIGURE 3:**
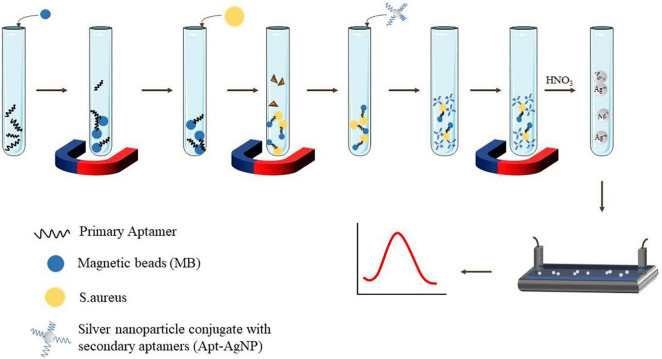
Schematic display electrochemical dual aptamer sandwich detection of *S. aureus*.

Voltammetry is a method of measuring current by controlling potential. Recently, Abbaspour et al. (2015) employed a sandwich structure modified with silver nanoparticles and aptamers to detect *S. aureus*. The primary aptamer was securely fixed to the magnetic bead in order to catch *S. aureus*, while the secondary aptamer is combined with silver nanoparticles to improve the specific electrochemical properties. In addition, the LOD of this voltammetry was shown to be 1.0 CFU/ml, and the dynamic range was 10–1 × 10^6^ CFU/ml (Abbaspour et al., 2015). Figure 3 shows the schematic of the classical two-aptamer sandwich method for the electrochemical detection of *S. aureus*. The construction of electrochemical aptasensors is not only applied to the risk assessment of *S. aureus* in food, but also has a good application in the risk assessment of toxins. Mousavi Nodoushan et al. (2019) detected SEB on screen-printed electrodes modified with graphene oxide (rGO) and nano-gold sea urchins (Aunus). DNA chain probes were connected to aptamers and probes were connected to Aunus electrodes. When the SEB appeared, the aptamer disconnected from the electrode and the peak current is recorded using an electrochemical signal generator. The electrochemical aptasensor developed is highly sensitive in milk, meat, and serum samples. The aptasensor had a LOD of 0.21 fM, with a wide linear range of 5.0–500.0 fM (Mousavi Nodoushan et al., 2019).

#### Aptasensor Detection Based on Impedimetry

Electrochemical impedance spectroscopy (EIS) is a technique used to study electrode systems and to conclude the quantification of electrochemical processes. Despite the complexity of EIS, it has high commercial potential, and has been widely used in environmental monitoring, disease monitoring, and other fields. One of its advantages is small-amplitude homeostatic disturbance, such that it can be realized without damaging the detection of the case. It may also measure, in case of uncertainty, the presence of REDOX pairs (Bahadır and Sezgintürk, 2014). The precision and operation procedure of the instrument has a certain influence on subsequent results. Zhang et al. (2019) used aptamer-magnetic separation in the resistivity method to detect *S. aureus*. The linear ranges of *S. aureus* were 4.1 × 10^3^ to 4.1 × 10^8^ CFU/ml, and LOD was 4.0 × 10^3^ CFU/ml in pure water (Zhang et al., 2019). Reich et al. (2017), who combined EIS use with aptamers to detect *S. aureus*, showed a LOD of 10 CFU/ml. EIS aptasensors also have many applications in the risk assessment of toxins in food. Combining induced release strategy with amplification of HCR signals, Chen et al. (2019) developed an electrochemically competitive nanoprobe for ultrasensitive specificity SEB detection measured with chronocoulometry. Three classical electrodes were used for this experiment, namely, a modified gold electrode, an Ag/AgCl reference electrode, and an auxiliary platinum wire electrode. Under the first-rank conditions, the charge difference of SEB increased linearly with the logarithmic increase of SEB concentration in the range of 5 pg/ml to 100 ng/ml, and the detection limit was as low as 3 pg/ml (Chen et al., 2019).

## Conclusion

This article reviews how aptasensors have been applied to risk assessment in food, especially for foodborne pathogens such as *S. aureus*. As a tool of risk assessment of foodborne pathogens, aptasensors have good competitiveness in terms of time, sensitivity, specificity, and cost.

**FIGURE 4:**
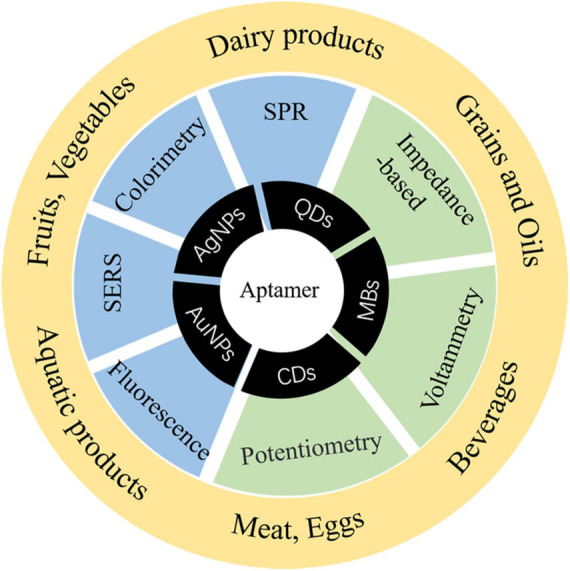
Applicable materials, methods, and applications of aptamers for *S. aureus* risk assessment.

*Staphylococcus aureus* produces enterotoxin (especially in animal-derived food such as milk and cream, which are easily infected) to cause food poisoning and has become a worldwide foodborne pathogenic factor. It is necessary to establish a method with less time and high sensitivity for *S. aureus*. It is commonly known that aptasensor detection has been widely applied to foodborne pathogens. However, the sensibility is limited, and it is still the focus of future research to improve the sensitivity and shorten the detection time of foodborne pathogens. In the practical application of aptasensors, there are many problems that need to be solved, for example, finite configuration of aptamers, aptamers with a high negative charge are difficult to combine with a negatively charged target, time-consuming process, and low success rate of SELEX. In the next step, how to quickly obtain excellent aptamers, shorten the specific aptamer screening cycle, improve the success rate of SELEX, and save cost and investment will become a research focus, so as to further promote the application of aptamer technology in the detection of pathogens. In the future, it is hoped that aptamers combined with a variety of nanomaterials to form simpler and faster aptasensors for detection will not only improve the success rate of detection but also provide a variety of data processing methods in different types of food. Figure 4 shows the applications of materials for *S. aureus* aptasensor detection in food risk assessment. By virtue of its remarkable detection characteristics, simultaneously solving the above-mentioned issues, detection methods are prone to false-positive results and other defects, and there is thus room for the further development of aptamers. New technologies have also been mentioned in the paper, including new applications of signal transduction and the combination of signal transduction and enhancement of signal amplification mode. With these advances, the sensitivity and time of detection have greatly improved. It is predicted that aptamers will be further combined with nanomaterials, and portable detection instruments will be developed.

## Author Contributions

ZH and XY drafted the manuscript. WW, QY, and YZ designed the concept and revised the manuscript. All authors contributed to the article and approved the submitted version.

## Conflict of Interest

The authors declare that the research was conducted in the absence of any commercial or financial relationships that could be construed as a potential conflict of interest.

## Publisher’s Note

All claims expressed in this article are solely those of the authors and do not necessarily represent those of their affiliated organizations, or those of the publisher, the editors and the reviewers. Any product that may be evaluated in this article, or claim that may be made by its manufacturer, is not guaranteed or endorsed by the publisher.
